# Acute Effects of Air Pollution on Pulmonary Function, Airway Inflammation, and Oxidative Stress in Asthmatic Children

**DOI:** 10.1289/ehp11813

**Published:** 2008-11-28

**Authors:** Ling Liu, Raymond Poon, Li Chen, Anna-Maria Frescura, Paolo Montuschi, Giovanni Ciabattoni, Amanda Wheeler, Robert Dales

**Affiliations:** 1 Healthy Environments and Consumer Safety Branch, Health Canada, Ottawa, Ontario, Canada;; 2 Department of Pharmacology, Faculty of Medicine, Catholic University of the Sacred Heart, Rome, Italy

**Keywords:** air pollution, asthma, children, exhaled breath condensate, inflammation, oxidative stress, pulmonary function

## Abstract

**Background:**

Air pollution is associated with respiratory symptoms, lung function decrements, and hospitalizations. However, there is little information about the influence of air pollution on lung injury.

**Objective:**

In this study we investigated acute effects of air pollution on pulmonary function and airway oxidative stress and inflammation in asthmatic children.

**Methods:**

We studied 182 children with asthma, 9–14 years of age, for 4 weeks. Daily ambient concentrations of sulfur dioxide, nitrogen dioxide, ozone, and particulate matter ≤ 2.5 μm in aerodynamic diameter (PM_2.5_) were monitored from two stations. Once a week we measured spirometry and fractional exhaled nitric oxide (Fe_NO_), and determined thiobarbituric acid reactive substances (TBARS) and 8-isoprostane—two oxidative stress markers—and interleukin-6 (IL-6) in breath condensate. We tested associations using mixed-effects regression models, adjusting for confounding variables.

**Results:**

Interquartile-range increases in 3-day average SO_2_ (5.4 ppb), NO_2_ (6.8 ppb), and PM_2.5_ (5.4 μg/m^3^) were associated with decreases in forced expiratory flow between 25% and 75% of forced vital capacity, with changes being −3.1% [95% confidence interval (CI), −5.8 to −0.3], −2.8% (95% CI, −4.8 to −0.8), and −3.0% (95% CI, −4.7 to −1.2), respectively. SO_2_, NO_2_, and PM_2.5_ were associated with increases in TBARS, with changes being 36.2% (95% CI, 15.7 to 57.2), 21.8% (95% CI, 8.2 to 36.0), and 24.8% (95% CI, 10.8 to 39.4), respectively. Risk estimates appear to be larger in children not taking corticosteroids than in children taking corticosteroids. O_3_ (5.3 ppb) was not associated with health end points. Fe_NO_, 8-isoprostane, and IL-6 were not associated with air pollutants.

**Conclusion:**

Air pollution may increase airway oxidative stress and decrease small airway function of asthmatic children. Inhaled corticosteroids may reduce oxidative stress and improve airway function.

Asthma is one of the most prevalent chronic health conditions in Canadian children. The 1998–1999 National Population Health Survey found that the prevalence of physician-diagnosed asthma is 8.4% among the whole population and 10.7% among children and teenagers, corresponding to approximately 845,000 children (Canadian Institute for Health Information et al. 2001). Asthma compromises the quality of life for patients and their families and imposes a burden on the nation’s health care expenditure. In 1990, the economic costs of asthma for Canada were estimated to fall between $504 and $648 million (Krahn et al. 1996).

Epidemiologic studies have shown that air pollution is associated with asthma-related increases in hospital admissions (Burnett et al. 1999; Wong et al. 2001), emergency department visits (Stieb et al. 2000; Sunyer et al. 1997; Villeneuve et al. 2007), and respiratory symptoms (Brauer et al. 2002; Vichit-Vadakan et al. 2001), and decreases in pulmonary function (Delfino et al. 2004; Gielen et al. 1997). However, there is a dearth of information about whether these associations can be attributed to lung tissue injury in an asthmatic population, partly because of the lack of appropriate non-invasive biomarkers indicating lung inflammation and tissue injury. In recent years, some researchers have used fractional exhaled nitric oxide (Fe_NO_) to investigate the relationship between exposure to air pollutants and airway inflammation (Delfino et al. 2006; Mar et al. 2005).

Measurements of mediators in exhaled breath condensate have been used to better understand the pathological mechanisms of airway diseases such as asthma, chronic obstructive pulmonary disease, and cystic fibrosis (Horvath et al. 2005; Montuschi 2005). Recently, analysis of mediators in condensed breath has been extended to include lung diseases caused by occupational exposure (Lehtonen et al. 2007; Pelclová et al. 2007). Asthma is a chronic airway inflammatory disease involving a variety of inflammatory cell types and proinflammatory mediators. Reactive oxygen species are also thought to play an active role in inducing airway inflammation and hyperresponsivness (Hulsmann et al. 2007). Reactive oxygen species result from an increased burden of exogenous oxidants, endogenous lipid peroxidation, as well as from inflammatory cells (Halliwell and Gutteridge 1989). The level of reactive oxygen species in the airways can be determined by assaying concentrations of oxidative stress markers in breath condensate (Horvath et al. 2005).

We designed this longitudinal study to test the hypothesis that an acute increase in ambient air pollutants is associated with increased levels of markers of oxidative stress and inflammation in breath condensate of children with asthma, and that changes of breath markers are consistent with changes in pulmonary function and Fe_NO_.

## Materials and Methods

### Study subjects

The study protocol was approved by the Research Ethics Board of Health Canada. The parent of each child gave written informed consent before the child’s participation in this study.

The study was conducted between October and December of 2005 in Windsor, Ontario, Canada. Asthmatic children, 9 to 14 years of age, from nonsmoking households, were identified from a questionnaire survey of grades one to eight of elementary schoolchildren whose parents were asked whether or not the child had doctor-diagnosed asthma. The parents who gave a positive response were telephoned to confirm the child’s current status of doctor-diagnosed asthma and whether there was any smoking of tobacco products in the household. One hundred eighty two subjects agreed to participate in this longitudinal study. Information on family income and family asthma history was extracted from the survey questionnaire. For logistical purposes, children were studied in two test periods, either between 11 October and 11 November (95 children), or between 14 November and 9 December 2005 (87 children). We did not advise parents to alter their children’s daily activity and the use of asthma medications.

### Ambient air pollution monitoring

**Figure 1:**
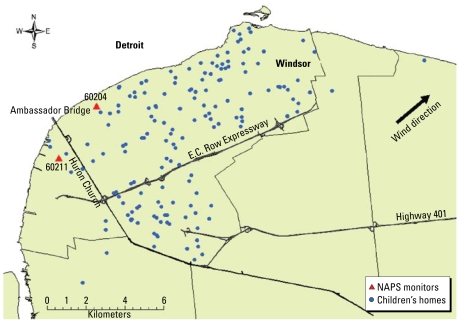
Map of Windsor, Ontario, Canada.

Hourly measures of particulate matter ≤ 2.5 μm in aerodynamic diameter (PM_2.5_), sulfur dioxide, nitrogen dioxide, and ozone were available from the National Air Pollution Surveillance network of Environment Canada (http://www.etc-cte.ec.gc.ca/NapsData). There were two monitoring stations representing Windsor, located upwind from the prevailing winds. Of 182 participating children, 180 of them lived within a 10-km radius from the air monitoring sites, and one lived 12 km and another 17 km from the air sampling sites (Figure 1). Values from these two monitors were averaged. SO_2_ was measured using ultraviolet fluorescence and the electrical conductivity from changes in chemical composition of a bromine solution, NO_2_ measured using chemiluminescence, O_3_ measured using ultraviolet absorbance, and PM_2.5_ measured using tapered element oscillating microbalance monitor. Pollutant concentrations were averaged over a 24-hr period.

### Measurement of spirometry and Fe_NO_

Once a week, registered respiratory technologists visited the children’s schools, and conducted tests of spirometry and Fe_NO_ and collected exhaled breath condensate on study participants. These tests were run on the same day of the week for each child.

Spirometry was performed according to American Thoracic Society criteria (American Thoracic Society 1995). KoKo Spirometers (Ferraris CardioRespiratory; Pulmonary Data Services, Inc. Louiseville, CO, USA) were calibrated daily and results adjusted for temperature and barometric pressure. A maximum of eight forced vital capacity (FVC) maneuvers were carried out in an attempt to achieve three acceptable flow-volume loops and two being within 0.20 L for FVC and forced expiratory volume in one second (FEV_1_), and within 0.20 L/sec for the mean forced expiratory flow between 25% and 75% of the FVC (FEF_25–75%_). The value assigned to the child was the largest acceptable value within 200 mL of a second value.

Single-breath on-line measures of Fe_NO_ were carried out according to American Thoracic Society and European Respiratory Society Guidelines (American Thoracic Society 2005) using an Eco Physics CLD AL MED chemiluminescence analyzer (Eco Medics AG, Duernten, Switzerland). Before performing a slow vital capacity maneuver over at least 6 sec at 0.05 L/sec, subjects took three tidal volume breaths through a DENOX 88 (Eco Medics AG, Duernten, Switzerland) which contains an NO_x_ absorber cartridge to scrub the ambient air to a value of between 0 and 1.5 ppb. The test was repeated a maximum of eight times in an attempt to obtain at least two acceptable plateau Fe_NO_ values within 10%. The value assigned to the subject was the mean of the two values. We calculated Fe_NO_ statistics using spiroWare 88 software (Eco Medics AG, Duernten, Switzerland).

### Collection of exhaled breath condensate, and measurement of amylase, oxidative stress, and inflammation

Exhaled breath condensate was collected using an RTube (Respiratory Research Inc., Charlottesville, VA, USA). Participants sat and breathed at tidal volumes orally into a mouthpiece attached to a cold condenser, for 10 min. Approximately 1 mL of breath condensate was collected. The condensed breath was then transferred into a microtube and stored at −20°C and then −80°C until use.

To determine saliva contamination, we measured α-amylase activity in breath condensate using an assay kit (Pointe Scientific, MI, USA), which follows the increase in absorbance at 405 nm as the artificial substrate 2-chloro-*p*-nitrophenyl-α-d-maltotrioside is hydrolyzed.

Thiobarbituric acid reactive substances (TBARS) were determined in duplicate by a fluorescence method adapted from Yagi (1982). We used malonaldehyde bis-(dimethyl acetal) (Aldrich Chemical Co., Milwaukee, WI, USA) to construct a standard curve. To reduce the background interference with the very low levels of TBARS present in exhaled breath condensates, all glassware was washed in soap-free detergent followed by rinsing with distilled water and ethanol before use. We used butanol (OmniSolv, 99.95%; EM Science, Darmstadt, Germany) for extraction. The *n*-butanol extract was transferred to a Whatman glass bottom microplate (Whatman, NJ, USA), previously washed with ethanol, and the fluorescence measured at 530 nm/590 nm, excitation/emission, with a Cytofluor 2350 reader (Millipore, Billerica, MA, USA). The detection limit was 0.02 μM (three times the standard deviation above the blank). The within-run coefficient of variation was 8%.

A colorimetric enzyme immunoassay kit (Cayman Chemical, Ann Arbor, MI, USA) was used to measure 8-isoprostane concentrations in breath condensate. The assays were done in triplicate. A modified standard curve for 8-isoprostane was established for concentrations between 0.975 and 62.5 pg/mL. We were able to detect 8-isoprostane at a concentration as low as 0.975 pg/mL (three times the standard deviation above the blank). The within-run coefficient of variation was 5%. We also measured 8-isoprostane on 8 random samples using radioimmunoassay, a procedure previously described by Montuschi et al. (2003), and compared the results from both methods [see Supplemental Material, Table 1 (available online at http://www.ehponline.org/members/2008/11813/suppl.pdf)]. Pearson correlation coefficient for results from two methods was 0.744 (*p* = 0.055), with a mean positive bias of approximately 35% in favor of the radioimmunoassay. Such a difference may be attributed to variations in the avidity and specificity of the antibodies used and the inherent variation during extrapolation from calibration curves at very low levels that were typical of exhaled breath condensates.

We determined the level of interleukin-6 (IL-6) using an ELISA kit from R&D Systems (Minneapolis, MN, USA). We conducted the ELISA assays in duplicate by following manufacturer’s instructions accompanying the kit. The detection limit was 0.48 pg/mL.

### Statistical analysis

We analyzed the correlations among environmental factors, daily use of inhaled corticosteroids (ICS) or short-acting β-agonist (SABA), and testing period, using Spearman rank order correlation, because some variables were not normally distributed. We used mixed-effects regression models to account for repeated measures and to analyze the associations between pollutants and health end points, assuming random slope and random intercepts, and accounting for intraindividual correlation using S-PLUS (version 6.1; Insightful Corporation, Seattle, WA, USA). Data for Fe_NO_, TBARS, and 8-isoprostane were log-transformed to fit a normal distribution. Because > 99% of the samples gave a value > 0 for TBARS and 8-isoprostane, and most of them were above the detection limit, data points below the detection limit were not given special treatment. For exposure variables, we tested models for multiday moving averages to examine potentially delayed response. Models were adjusted for time-dependent covariates that were statistically associated with either the exposure or response. We adjusted for testing period (as an indicator variable) to reduce the impact of seasonality. We also included product terms for the interaction of pollutant concentrations with sex and with the use of ICS to test potential modifying effects of sex and ICS. Since subjects served as their own control, we did not adjust for factors which remained constant during the study period such as age, race, family income, and family asthma history. Because health testing was kept on the same day of week for each child, we did not adjust for the effect of day-of-week in the models.

When interactions were detected between pollutants and sex or the use of ICS, the participants were stratified for further analyses. We also ran two-pollutant models to adjust for potential confounding effects of co-pollutants. Results were expressed as percent change in FEV_1_, FEF_25–75%_, Fe_NO_, TBARS and 8-isoprostane per increase in inter-quartile-range concentration of a pollutant. A two-tailed value of *p* ≤ 0.05 was considered statistically significant.

## Results

A total of 182 asthmatic children participated in the study, for a total of 691 spirometry test results, 672 Fe_NO_ test results, and 616 breath samples. Seven children had 5 measurement days, 142 had 4 days, 27 had 3 days, and 6 had 2 days. In total, there were 39 measurement days during the study period, and no missing day for air monitoring to match health testing days. The characteristics of this cohort are presented in Table 1. We measured the concentration of amylase in 25% of breath samples to determine saliva contamination. The median amylase concentration was 0.60 unit/L, with the 95th percentile being 1.46 unit/L. None of the samples had amylase concentrations near the reported salivary amylase concentration of 1,250 unit/L (Brand et al. 2006). We estimated that saliva contamination was likely < 0.1%, and therefore interference of breath analysis by saliva was negligible. In 616 breath samples, only 2 samples had detectable IL-6 levels. We therefore did not conduct further analysis on IL-6.

The environmental conditions and daily concentrations of air pollutants measured during the study period are presented in Table 2. Because Spearman rank order correlation analyses showed that daily temperature, relative humidity and testing period were significantly correlated with some of the pollutants (Table 3), we included these variables in models to adjust for any confounding/modifying effects. We also included daily use of SABA and ICS to adjust for their potential confounding effects. Because pollutants were correlated with each other (Table 3), we also ran two-pollutant models to adjust for confounding.

### Results from single-pollutant regression models

Table 4 presents percentage changes of health end points in relation to an increase in interquartile range concentrations of pollutants in a single-pollutant model which also included interaction terms of ICS and sex with pollutants. FEV_1_ and FEF_25–75%_ exhibited a consistent trend of negative associations with all four pollutants, with FEF_25–75%_ having a statistically significant association with NO_2_ and PM_2.5_. The significant associations for FEF_25–75%_ persisted for at least 3 days. Fe_NO_ had a trend of positive associations with SO_2_, NO_2_, and PM_2.5_, but the associations were not statistically significant. Fe_NO_ had a statistically significant negative association with O_3_. TBARS was significantly positively associated with SO_2_, NO_2_, and PM_2.5_, but not with O_3_. The associations for TBARS persisted for at least 3 days. 8-Iso-prostane was significantly positively associated with same-day SO_2_, but not associated with other pollutants.

### Modifying effects by the use of ICS and sex

**Figure 2:**
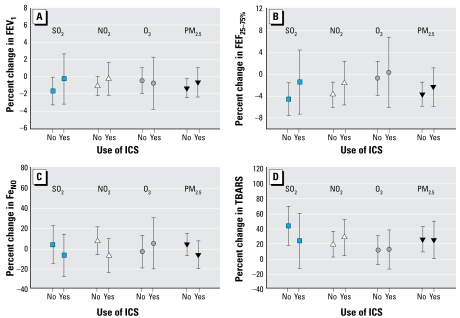
Percent changes and 95% confidence intervals in FEV_1_ (*A*), FEF_25–75%_(*B*), Fe_NO_ (*C*), and breath TBARS (*D*) of asthmatic children taking or not taking ICS during the study period, in association with an interquartile-range increase in a pollutant. Sixty-eight asthmatic children took (Yes), and 114 children did not take (No) ICS. Pollutant concentrations were averaged over 3 days including the same day and 2 days before the clinical testing was done. Models were adjusted for testing period, daily temperature, relative humidity, and use of SABA.

Product terms between pollutants and the use of ICS showed significant interactions for some of the health end points. For example, the product term for SO_2_ and ICS was positively associated with TBARS (*p* = 0.04), but negatively associated with FEV_1_ (*p* < 0.01), FEV_25–75%_ (*p* = 0.02), and 8-isoprostane (*p* = 0.01), indicating that the use of ICS was a significant effect modifier. The participants were then stratified by the use of ICS during the study period to adjust for modifying effects of use of ICS (Figure 2). Children who did not use ICS appeared to have larger risk estimates for pollutants than did those who used ICS. Among children not taking ICS, risk estimates for pollutants were statistically significant with respect to FEV_1_, FEF_25–75%_, and TBARS. Risk estimates for Fe_NO_ and 8-isoprostane were not very different between the two groups of children (data not shown).

Product terms for pollutants and sex showed no significant interaction (*p* > 0.05), suggesting that sex was not a significant modifier for the associations between air pollutants and health end points. We therefore did not run further analysis on boys and girls separately.

### Results from two-pollutant models

**Figure 3:**
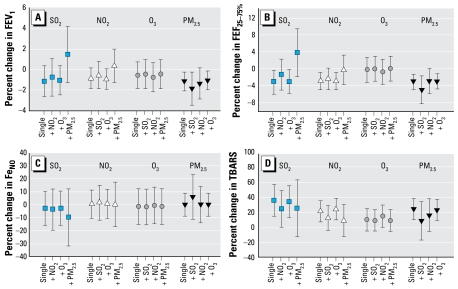
Percent changes and 95% confidence intervals in FEV_1_ (*A*), FEF_25–75%_(*B*), Fe_NO_ (*C*), and breath TBARS (*D*) of asthmatic children, in association with an interquartile-range increase in a pollutant, in one- and two-pollutant models. Pollutant concentrations were averaged over 3 days including the same day and 2 days before the clinical testing was done. Models were adjusted for testing period, daily temperature, relative humidity, and use of SABA and ICS.

Because we found consistent associations with pollutants for FEV_1_, FEF_25–75%_, Fe_NO_, and TBARS, we ran two-pollutant models on these end points to adjust for confounding effects of co-pollutants (Figure 3). For FEV_1_ and FEF_25–75%_, although two-pollutant models did not result in marked changes in risk estimates for PM_2.5_ and O_3_, including PM_2.5_ in models did somewhat change risk estimates for SO_2_ and NO_2_, suggesting that the effects of SO_2_ and NO_2_ on pulmonary function were influenced by PM_2.5_. For Fe_NO_ and TBARS, including two pollutants in models did not markedly change the regression results, suggesting that the results of each pollutant were independent of other pollutants.

## Discussion

Principal findings of this study are that SO_2_, NO_2_, and PM_2.5_ in ambient air were significantly associated with decrements in FEV_1_ and FEF_25–75%_, and increases in TBARS in breath condensate among children with asthma. The risk estimates were larger for pollutant concentrations averaged over 3 days than over a shorter term. Clinically FEF_25–75%_ has been considered to be sensitive in detecting small airway dysfunction (Lebecque et al. 1993). These associations were robust after inclusion of daily temperature, relative humidity, study period, and the use of asthma medications SABA and ICS in models. The associations for O_3_ and PM_2.5_ were not sensitive to inclusion of co-pollutants, as adjusting for other pollutants in the model did not markedly change the strength of the risk estimates for O_3_ and PM_2.5_. The associations between pulmonary function decrements and SO_2_ and NO_2_ were sensitive to the inclusion of PM_2.5_ in models, suggesting that PM_2.5_ may have confounded some of the effects of SO_2_ and NO_2_. Adverse changes in FEF_25–75%_ were consistent with those of TBARS in response to SO_2_, NO_2_, and PM_2.5_ with a similar lag structure, suggesting a coherent outcome for small airway function and oxidative stress. Our results on pulmonary function changes were similar to those published previously (Delfino et al. 2004; Epton et al. 2008; Gielen et al. 1997; Romieu et al. 2008), although air pollutant levels in the present study were low compared to those studies.

Exhaled breath condensate has been used in clinical settings to study pathological mechanisms of respiratory illness such as asthma, chronic obstructive pulmonary disease, and cystic fibrosis (Baraldi et al. 2003; Horvath et al. 2005). Elevated concentrations of oxidant nitrate (Balint et al. 2001), IL-6 and leukotriene B4 (Carpagnano et al. 2003), and 8-isoprostane (Montuschi et al. 2000), have been found in breath condensate of adult smokers. Markers of inflammation and oxidative stress in breath were associated with occupational exposure to cobalt and tungsten (Goldoni et al. 2004), chromium (Caglieri et al. 2006), silica (Pelclová et al. 2007), and asbestos (Lehtonen et al. 2007). Montuschi et al. (2002) exposed healthy nonsmoking adults to high concentration of O_3_ (400 ppb) and clean air for 2 hr on separate occasions in a laboratory setting, and reported that exposure to O_3_ led to a significant increase in 8-isoprostane concentrations in breath condensate. Barregard et al. (2008) exposed adult human subjects to wood smoke in a controlled environment and reported an increase in malondialdehyde levels in breath for at least 20 hr postexposure. However, little work has been done to use biomarkers in breath condensate to examine the effects of ambient air pollutants on airway inflammation and oxidative stress among asthmatic children. Very recently, Epton et al. (2008) reported a cohort study on 12- to 18-year-old male healthy and asthmatic students in a New Zealand boarding school, where they found small effects of PM_10_ (PM with aerodynamic diameter < 10 μm) in ambient air on FEV_1_, but no effect on pH and hydrogen peroxide in exhaled breath. However, with a panel of asthmatic children living in Mexico City, Romieu et al. found an association between malondialdehyde in exhaled breath and PM_2.5_ and O_3_ in ambient air (Romieu et al. 2008). A 14.2-μg/m^3^ (interquartile-range) increase in 8-hr moving average PM_2.5_ was significantly associated with a 1.12-nmol increase in malondialdehyde, while a 15.9-ppb (inter-quartile-range) increase in 8-hr moving average O_3_ was significantly associated with a 1.16-nmol increase in malondialdehyde. Our results are consistent with these findings in that inhaled ambient air pollutants were significantly associated with an increase in an oxidative stress marker in breath condensate.

Oxidative stress–inducing reactive oxygen species are physiologically active mediators that can be induced by air pollutants. PM_2.5_, SO_2_, NO_2_, and O_3_ have been demonstrated to cause formation of excessive amount of reactive oxygen species in airways and in the cardiovascular system in experimental animals, leading to tissue inflammation and cell death (Dye et al. 1997; Meng et al. 2003; Persinger et al. 2002). Oxidative stress has been linked to clinical phenotypes such as asthma and atherosclerosis (Chuang et al. 2007; Walter et al. 2004). TBARS are a group of low-molecular-weight chemicals that are formed during the decomposition of lipid peroxidation products (Halliwell and Chirico 1993), and thus are often used as an index of lipid peroxidation and oxidative stress (Kodavanti et al. 2000; Rhoden et al. 2004; Walter et al. 2004). 8-Isoprostane is a prostaglandin F2α–like compound produced by free radicals via the nonenzymatic peroxidation of arachidonic acid in membrane phospholipids (Morrow et al. 1990). Because TBARS are derived from oxidative degradation of a broader range of biological substances including lipids and sugars than is 8-isoprostane (Halliwell and Gutteridge 1981; Janero 1990), and because we observed an increase in TBARS but not 8-isoprostane in breath samples in relation to elevated air pollutants, it appears that TBARS may be a more sensitive biomarker than 8-iso-prostane in breath condensate as a useful tool for investigating air pollution-related oxidative stress among asthmatic children, especially for acute exposures. It remains to be seen whether immunoassays with significantly higher sensitivity and specificity can improve the efficacy of 8-isoprostane as a biomarker of oxidative stress in exhaled breath condensate.

ICS are thought to reduce inflammation and oxidative stress in airways (Baraldi et al. 2003; Khor et al. 2007). Children with asthma exacerbation have reduced 8-isoprostane in breath condensate after ICS treatment (Baraldi et al. 2003). In the present study, we found that ICS seems to be able to reduce some of the adverse effects of air pollutants, as the risk estimates appear to be larger for the children who did not take ICS during the study period. Consistent with our findings, previous studies have also reported stronger associations between exposure to air pollutants and decrements in pulmonary function and increases in Fe_NO_ for children not taking anti-inflammatory medications than for children taking the medications (Delfino et al. 2006, 2008; Mar et al. 2005). In the present study only 37% of children used ICS, which could result in a reduced statistical power to detect associations with pollutants in this group.

Oxidative stress often occurs along with tissue inflammation, which induces the release of reactive oxygen species to cause tissue injury (Chuang et al. 2007; Dye et al. 1997; Kodavanti et al. 2000). Unlike previously published studies (Delfino et al. 2006; Mar et al. 2005), in our study we did not find statistically significant adverse changes in inflammatory marker Fe_NO_ associated with air pollution, and the level of IL-6 in breath condensate was too low to be detectable. One interpretation for the results of inflammation markers was that we might need a larger sample size to detect significant changes in airway inflammation. Another possible explanation might be that the inflammation in lower airways of the children was not captured by Fe_NO_ measured at a flow rate of 0.05 L/sec; Barregard et al. (2008) reported that exposure to wood smoke particles resulted in significant increase in Fe_NO_ measured at a flow rate of 0.27 L/sec, but not at 0.05 L/sec. Alternatively, this group of asthmatic children might have been in such a state of airway inflammation that it overwhelmed the effects of air pollution, particularly when exposure concentrations were lower than those in other studies (Delfino et al. 2006; Mar et al. 2005).

We did not find a consistent adverse effect of O_3_. O_3_ exhibited a significantly negative association with Fe_NO_ (Table 4), but a positive association with TBARS (Figure 3). The negative association between O_3_ concentration and Fe_NO_ is counterintuitive, because laboratory studies have shown that O_3_ at high concentrations (200–400 ppb) caused inflammation in the airways of human subjects (Balmes et al. 1996; Frampton et al. 1997). In the present study, adjusting for co-pollutants and study periods in the models did not change the results. A sound interpretation for this result remains to be found at this point in time.

When a large number of regression models are run on a data set, some statistically significant associations may occur by chance. To reduce the possibility of spurious relationships due to multiple comparisons, we limited sensitivity analyses to those that consistently demonstrated statistical significance in base models, and considered only results showing a consistent pattern as actual effects.

## Conclusion

Results from our study demonstrate a significant decrement in small airway function and an increase in airway oxidative stress in asthmatic children in association with SO_2_, NO_2_, and PM_2.5_ in ambient air. TBARS in breath condensate may be a useful tool for investigating air pollution-related oxidative stress among asthmatic children. ICS seems to be able to reduce some of the adverse respiratory response to air pollutants.

## Figures and Tables

**Table 1:** Selected characteristics of 182 asthmatic children from Windsor, Ontario, Canada.

Characteristic	Value
Age (years) [median (range)]	11 (9–14)
Sex [no. (%)]	
Female	68 (37.4)
Male	114 (62.6)
Race [no. (%)]	
Caucasian	160 (87.9)
Other	22 (12.1)
Parental asthma [no. (%)]	
No	11 (6.0)
Yes	70 (38.5)
Unknown	101 (55.5)
Self-reported medication use^a^ [no. (%)]	
ICS	68 (37.4)
SABA	64 (35.2)
Other asthma medications	33 (18.1)
None	77 (42.3)
FEV_1_ (L) [median (5th– 95th percentile)]	2.4 (1.7–3.3)
Percent predicted FEV_1_ (%) [median (5th– 95th percentile)]^b^	90.5 (68.6–107.1)
FEF_25–75%_ (L/sec) [median (5th– 95th percentile)]	2.5 (1.3–3.9)
Percent predicted FEF_25–75%_ (%) [median (5th– 95th percentile)]^b^	78.2 (45.3–118.0)
Fe_NO_ (ppb) [median (5th– 95th percentile)]	19.4 (4.5–81.6)
TBARS (μM) [median (5th– 95th percentile)]	0.26 (0.03–1.53)
8-Isoprostane (pg/mL) [median (5th– 95th percentile)]	1.43 (0.30–4.20)

a More than one medication could be used.

b Predicted normal values were calculated based on a given height, age, sex, and race/ethnicity (Hankinson et al. 1999).

**Table 2:** Values for selected environmental conditions during study period.

	Median [interquartile range (5th–95th percentile)]		
Environmental variable	1-day average	2-day average	3-day average
SO_2_ (ppb)	4.5 [6.5 (0.5 to 15.5)]	5.0 [5.6 (1.0 to 13.0)]	5.6 [5.4 (1.3 to 13.8)]
NO_2_ (ppb)	19.8 [9.8 (9.5 to 29.5)]	18.3 [9.0 (11.3 to 28.0)]	18.3 [6.8 (12.3 to 27.0)]
O_3_ (ppb)	13.0 [9.0 (6.5 to 26.5)]	14.1 [6.4 (6.8 to 23.3)]	14.0 [5.3 (7.5 to 21.0)]
PM_2.5_ (μg/m^3^ )	6.5 [6.0 (2.0 to 19.0)]	6.6 [6.4 (2.0 to 15.0)]	6.9 [5.4 (2.7 to 14.3)]
Temperature (°C)	6.6 [13.0 (−7.7 to 15.4)]	7.4 [12.0 (−5.5 to 14.7)]	7.4 [11.2 (−5.9 to 14.5)]
Relative humidity (%)	67.4 [11.6 (53.3 to 87.9)]	65.6 [9.7 (54.4 to 75.6)]	65.7 [7.3 (54.9 to 74.2)]

Air quality was monitored between 8 October and 9 December 2005, 62 sampling days.

**Table 3:** Spearman rank order correlation coefficients of variables.

Variable	Temperature	Relative humidity	SO_2_	NO_2_	O_3_	PM_2.5_	SABA	ICS
Testing period	−0.73*	0.01	0.35*	−0.30*	0.06	−0.07	0.10*	−0.07
Temperature		0.18*	−0.17*	0.00	0.18*	0.10*	−0.10*	−0.02
Relative humidity			−0.04	0.03	−0.51*	0.20*	0.01	−0.02
SO_2_				0.18*	−0.02	0.56*	0.04	0.05
NO_2_					−0.51*	0.71*	−0.04	0.07
O_3_						−0.41*	0.00	−0.10*
PM_2.5_							−0.05	0.07
SABA								0.27*

* *p* < 0.05.

**Table 4:** Percent change (95% confidence interval) in health end points of 182 asthmatic children, in association with an interquartile-range increase of a pollutant, in single–pollutant models with various lag times.

Air pollutant	FEV_1_	FEF_25–75%_	Fe_NO_	TBARS	8–Isoprostane
SO_2_					
Same day	−0.3 (−1.3 to 0.7)	−1.0 (−3.1 to 1.3)	5.8 (−5.0 to 17.8)	17.4 (0.3 to 37.4)*	14.1 (2.5 to 26.9)*
Lag 1 day	0.1 (−1.1 to 1.2)	−0.9 (−3.1 to 1.4)	1.6 (−9.3 to 13.9)	14.1 (−1.8 to 32.5)	−2.4 (−11.7 to 7.9)
2-day average	−0.1 (−1.4 to 1.3)	−1.8 (−4.7 to 1.1)	5.9 (−9.3 to 23.7)	35.1 (9.5 to 66.8)*	9.4 (−5.2 to 26.2)
3-day average	−0.3 (−1.9 to 1.4)	−2.3 (−5.5 to 1.0)	1.7 (−14.1 to 20.3)	61.8 (24.9 to 109.5)*	−0.3 (−16.4 to 18.9

NO_2_					
Same day	−0.6 (−1.6 to 0.3)	−2.4 (−4.3 to −0.4)*	8.2 (−2.9 to 20.6)	21.2 (1.9 to 44.2)*	0.0 (−10.7 to 12.1)
Lag 1 day	−0.3 (−1.1 to 0.6)	−1.4 (−3.1 to 0.3)	3.7 (−6.1 to 14.6)	10.2 (−5.6 to 28.7)	−5.6 (−14.8 to 4.5)
2-day average	−0.6 (−1.6 to 0.4)	−2.4 (−4.3 to −0.3)*	7.4 (−4.6 to 21.0)	20.3 (−0.4 to 45.3)	−4.7 (−1.1 to 8.1)
3-day average	−0.8 (−1.9 to 0.3)	−2.8 (−5.0 to −0.5)*	0.5 (−12.1 to 14.9)	32.9 (7.2 to 64.6)*	−4.0 (−16.3 to 10.2)

O_3_					
Same day	−0.4 (−1.6 to 0.7)	0.2 (−2.1 to 2.5)	−12.2 (−22.3 to −0.8)*	5.0 (−13.2 to 27.0)	7.0 (−6.5 to 22.4)
Lag 1 day	−0.2 (−1.1 to 0.7)	0.1 (−1.7 to 1.9)	−8.3 (−16.0 to 0.2)	7.3 (−6.7 to 23.4)	6.6 (−3.4 to 17.6)
2-day average	−0.4 (−1.6 to 0.9)	0.4 (−2.2 to 3.0)	−16.0 (−26.4 to −4.1)*	14.4 (−8.5 to 43.0)	12.0 (−3.8 to 30.5)
3-day average	−0.5 (−2.0 to 1.0)	−0.4 (−3.5 to 2.7)	−1.6 (−16.7 to 16.1)	8.8 (−17.4 to 43.4)	15.9 (−3.5 to 39.1)

PM_2.5_					
Same day	−0.5 (−1.3 to 0.3)	−1.9 (−3.5 to −0.3)*	5.3 (−3.6 to 15)	16.9 (2.2 to 33.6)*	5.1 (−3.6 to 14.5)
Lag 1 day	−0.3 (−1.1 to 0.5)	−1.2 (−2.8 to 0.3)	1.7 (−6.3 to 10.4)	14.6 (0.8 to 30.4)*	−3.8 (−12.1 to 5.3)
2-day average	−0.6 (−1.5 to 0.4)	−2.0 (−3.8 to −0.2)*	4.3 (−5.4 to 15.1)	22.0 (4.8 to 42.1)*	0.1 (−9.8 to 11.1)
3-day average	−1.1 (−3.1 to 0.9)	−3.3 (−7.2 to 0.8)	−17.3 (−33.5 to 2.9)	69.1 (20.1 to 138.2)*	5.8 (−15.8 to 33.0)

2-day average: the median concentration of a pollutant collected on the day and 1 day before the clinical testing was done. 3-day average: the median concentration of a pollutant collected on the day and 2 days before the clinical testing was done. Models were adjusted for testing period, daily temperature, relative humidity, and use of SABA and ICS. Interaction terms were included in the models to test modifying effects of taking ICS (a binary indicator) and sex (binary indicator) on air pollutants.

* *p* < 0.05.
